# Gene expression during *Drosophila melanogaster *egg development before and after reproductive diapause

**DOI:** 10.1186/1471-2164-10-242

**Published:** 2009-05-24

**Authors:** Dean A Baker, Steven Russell

**Affiliations:** 1 Department of Genetics, University of Cambridge, Downing Street, Cambridge CB1 3QA, UK; 2 Cambridge Systems Biology Centre, Tennis Court Road, Cambridge CB2 1QR, UK

## Abstract

**Background:**

Despite the importance of egg development to the female life cycle in *Drosophila*, global patterns of gene expression have not been examined in detail, primarily due to the difficulty in isolating synchronised developmental stages in sufficient quantities for gene expression profiling. Entry into vitellogenesis is a key stage of oogenesis and by forcing females into reproductive diapause we are able to arrest oogenesis at the pre-vitellogenic stages. Releasing females from diapause allows collection of relatively synchronous developing egg populations and an investigation of some of the transcriptional dynamics apparent before and after reproductive diapause.

**Results:**

Focusing on gender-biased transcription, we identified mechanisms of egg development suppressed during reproductive dormancy as well as other molecular changes unique to the diapausing female. A microarray based analysis generated a set of 3565 transcripts with at least 2-fold greater expression in females as compared to control males, 1392 such changes were biased during reproductive dormancy. In addition, we also detect 1922 up-regulated transcriptional changes after entry into vitellogenesis, which were classified into discrete blocks of co-expression. We discuss some of the regulatory aspects apparent after re-initiation of egg development, exploring the underlying functions, maternal contribution and evolutionary conservation of co-expression patterns involved in egg production.

**Conclusion:**

Although much of the work we present is descriptive, fundamental aspects of egg development and gender-biased transcription can be derived from our time-series experiment. We believe that our dataset will facilitate further exploration of the developmental and evolutionary characteristics of oogenesis as well as the nature of reproductive arrest in *Drosophila*.

## Background

Large-scale gene expression studies in *Drosophilamelanogaster *provide important insights into the global relationships that exist between transcription and development [1,2]. Profiling the earliest stages of embryonic development through to late adulthood, for example, reveals that up to 90% of transcripts are regulated at some stage during the life-cycle [1]. The most highly conserved transcriptional components in the embryo often originate maternally and are utilized in developmental processes before zygotic mechanisms have been induced [3]. Oogenesis is a dynamic, highly regulated process, that depends on the correct developmental, environmental and nutritional cues for normal progression. Here, we perform an extensive analysis of the expression patterns apparent over a 72 hour period during egg development in *Drosophila*. Our approach facilitates investigation of dynamics through key transition points in oogenesis by focusing on transcript profiles obtained before and after entry into vitellogenesis.

Egg development in *Drosophila *is characterized by a process of nutrient supply to the oocyte via supporting cells [4]. The earliest stages of oogenesis occur in the germarium, where new egg chambers are produced from germ-line and somatic stem cells. Once egg chambers have left the germarium and entered the vitellarum, they comprise three cell types: the oocyte, nurse cells and somatic follicle cells. Processes within the vitellarium then concern further maturation of these cells, including mechanisms that require the synchronization of processes in the ovary and fat body for successful egg production.

The sequence of developmental events during oogenesis are believed to follow the same overall pattern in species from the holometabolous group of insects, although specializations in ovarian development are often apparent due to differences in the nutritional or ecological challenges faced by different species [5]. Such specializations include: a) the degree of ovarian development that occurs during pupal stages, b) ovariole number and growth rate, or c) number of eggs produced. Entry into vitellogenesis and the switch from early egg chamber development to more intensive oocyte growth and maturation is, however, recognized as a common control point for progression past early egg development. Vitellogenesis is characterized by a considerable increase in the synthesis of yolk proteins and other cytoplasmic constituents necessary for embryogenesis. Under adverse environmental conditions, ovarian arrest can be an important feature of egg development if continued reproductive effort has little chance of success.

While the induction and physiological state of insects that utilize a reproductive dormancy period are similar among species, the duration and depth of arrest can vary widely. For example, species that display a strong diapausing response initiate a series of hormonally mediated events to prepare and subsequently reduce rates of metabolism and reproduction [6]. Once the diapausing phase is entered it is not possible for normal activity to resume until the developmental program is completed, regardless of environmental conditions. In contrast, the response of species with a shallow diapause is to reduce growth and prevent the onset of vitellogenesis almost immediately when environmental cues change. This state, sometimes referred to as quiescence, can be rapidly reversed under favourable conditions with little delay [7].

When exposed to over-wintering conditions, reproductive dormancy is induced in *D. melanogaster *and oogenesis is arrested with egg chambers in a pre-vitellogenic state [8]. Females remain in this depressed developmental state for several weeks, after which, regardless of the environmental conditions, egg production resumes. At any stage however, it is possible to restart egg development by returning females to normal environmental conditions. In comparison to other species, these characters indicate a somewhat shallow and conditional physiological response to ovarian arrest. Despite this flexibility, *D. melanogaster *diapause is under control of the endrocrine system and circadian response. Seasonal fluctuations affecting the light-entrained pacemaker cells of the brain, triggering hormonal conditions necessary for diapause, with *D. melanogaster *displaying circadian induced reproductive arrest under laboratory conditions [8] and over environmental clines [9,10]. This physiological state can be terminated by transferring individuals from a short day to long day photoperiod [8] or by the application of hormones from the endocrine system [11-13].

The immediate hormonal basis for physiologically induced changes during diapause, in most cases, is the absence of Juvenile Hormone [6]. Juvenile Hormone regulates a number of early female reproductive events to stimulate pre-vitellogenic growth of egg chambers [14]. However, while responsible for controlling early reproductive development, in *D. melanogaster *the deposition of yolk proteins within oocytes and progression through the oogenic cycle is also coincident with increasing titers of ecydsteroids [12,13]. Ultimately there is a complex relationship between Juvenile Hormone, ecydsteroids and yolk protein production stemming from environmental cues that influence progression through the oogenic cycle.

Here, we use developmental arrest in *D. melanogaster *to generate a transcriptional time-course from pre-vitellogenic diapause, through vitellogenesis and into the last stages of egg development within the abdomen. We expect that genes displaying sex-biased transcriptional abundance are sex-biased in function, and by focusing on gender-biased expression within fly abdomens we investigate reproductive dormancy as well as other molecular changes unique to the diapausing female. Of particular interest are those genes activated immediately when egg development enters vitellogenesis. We further identify and discuss co-expressed genes regulated after diapause in light of the underlying site of expression, functions, maternal contribution and evolutionary conservation characteristic of these groups.

## Results and discussion

### Experimental design

**Figure 1:**
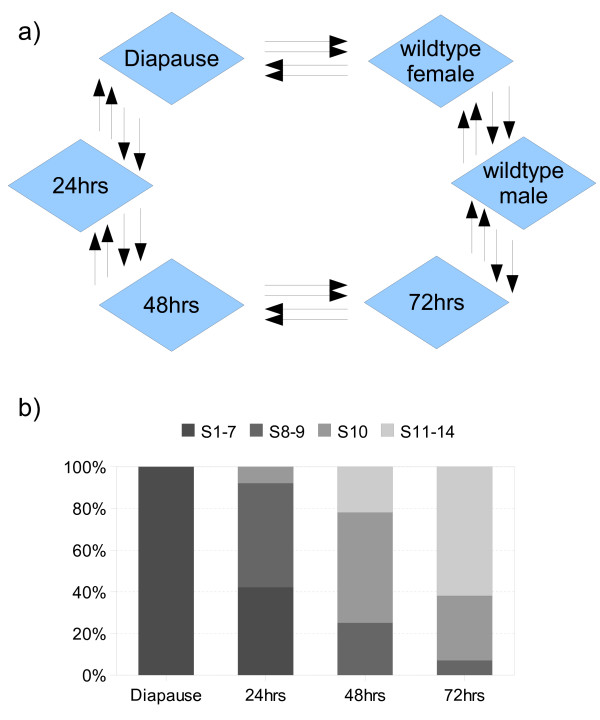
**Experimental design & ovary dissection**. **a) **Experimental design. Each arrow represents a separate biological replicate and indicates the Cy3 to Cy5 direction of dye swaps performed. **b) **Ovary dissection. Ovary dissection through the oogenic cycle and the relative proportion of ovarioles with maximum development in Stage 1–14 (n = 15).

Our goal was to explore the dynamics of transcriptional variation through the oogenic cycle in *Drosophila *by performing a genome-wide microarray analysis of gene expression during ovarian diapause and after the onset of vitellogenesis. Since tissues outside the ovary contribute to aspects of oogenesis, we elected to examine gene expression in dissected abdomens rather than just ovaries. The most efficient hybridization designs for two-colour microarray experiments involve combining samples in a staggered loop formation [15,16]. Time-points were collected before and after the release of reproductive arrest at 24 hour intervals and hybridized in a balanced loop design, including dye-swaps (Figure 1a). Eight biological replicates were performed for each treatment, including 3 day old male and female controls maintained under normal environmental conditions.

A short-day photoperiod (10:14 light:dark photoperiod) and moderate temperatures (11°C) are required for the induction of ovarian diapause [8]. For our microarray experiments, 0–4 hour post-eclosion females were immediately transferred to the restrictive environmental conditions. Females were subsequently collected for RNA extraction after 5–7 days, or were re-introduced to normal environmental conditions to continue egg development. A subset of ovaries were dissected from females at each time-point and the most mature stage of ovarioles counted to estimate progression through egg development (Figure 1b). All females dissected after 5–7 days in diapausing conditions were pre-vitellogenic. Egg development then progressed over the next 72 hrs after the release of diapause, with all ovarioles having entered vitellogenesis by 48 hrs and the maximum compliment of late stage eggs apparent at 72 hrs.

### Gender-biased transcription

A large proportion of genes in the genomes of sexually reproducing species display gender-biased expression (for a review see [17]). As a result of selection for traits that influence the fitness of each sex, males and females can differ dramatically, displaying sexual dimorphism in a range of physiological or behavioural characters [18-20]. Male and female genomes typically differ by only a small number of genes on the male Y chromosome, such that most differences between the sexes result from sex-specific developmental pathways that drive the differential expression of genes present in both sexes [21,22]. While dynamics of the transcriptional regulation responsible for gender-biased expression are of interest to developmental biologists, genes with sex-biased expression are also of interest to evolutionary biologists in light of the antagonistic selection patterns underlying such traits.

**Figure 2:**
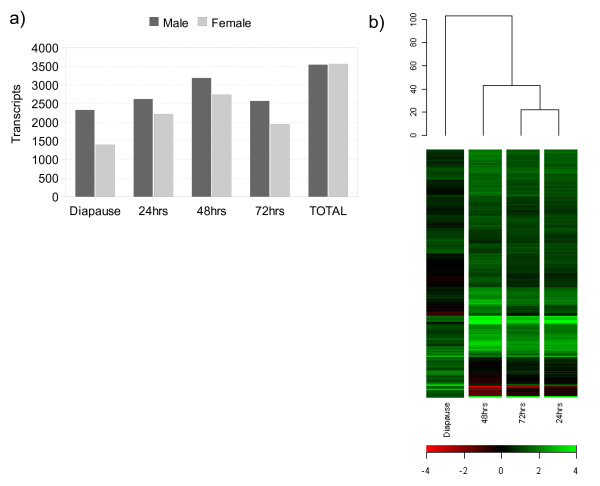
**Global patterns of gender-biased expression**. **a) **The ratio of gender-biased expression through the oogenic cycle. The Y-axis plots the number of transcripts biased in each sex according to each time point we examined. **b) **Hierarchical clustering of gender-biased similarity. Genes and samples were clustered across the female-biased dataset with centred gene expression data using Euclidean Distance to calculate similarity.

Adult males and females display widely divergent gene expression profiles, with over half of the genome displaying gender-biased regulation as a result of sex-specific physiology [23-25]. In the current study, comparison of a single time-point between male and control female samples indicate that at a 2-fold level, 2514 transcripts were female-biased and 3238 transcripts male-biased in expression. However, we also find that the number of genes with gender-biased expression varies significantly through the course of the oogenic cycle (q < 0.05, 2-fold; Figure 2a). Though typically in favour of greater male-biased transcription at any particular time-point, when transient expression is taken into account the total number of sexually dimorphic genes becomes nearly equal, representing 49% of the genome, in accordance with previous estimates of expression. The resulting set of female-biased genes indicates that 3565 transcripts have at least a 2-fold greater expression in the female compared to males: of these, 95% of transcripts in the control gender comparisons were represented [see Additional file 1]. We have ensured the statistical power of our experiment is appropriate for further analysis (See Methods), and the use of relatively stringent criteria allows us to focus on genes with robust expression changes. For those interested in more subtle changes in expression, our entire dataset is available from the public GEO repository.

The absolute number of genes reported as gender-biased in the literature varies widely, most probably due to a combination of different experimental platforms, statistical methods, strains or tissues used in each study. A consensus set of sex-biased *D. melanogaster *genes [26], derived from 3 independent microarray experiments [24,27,28], was also compared to our time-series analysis. Within the female-biased consensus set, 63% of genes were represented in our dataset (n = 338/538), whereas an additional 115 genes, while significantly female-biased (q < 0.05), were expressed at levels less than two-fold that of males. Such differences are, of course, not surprising and even in laboratory strains of *D. melanogaster *expression variation specific to females has been revealed [25]. Interestingly, 463 transcripts identified as unbiased in the consensus set were in fact up-regulated in females at some stage during the time-course. Certainly, time-series experiments are expected to reveal information about transient expression patterns that single-time point experiments cannot, especially for genes with transient up-regulated expression.

### Patterns of global gene regulation during egg development

At emergence, *D. melanogaster *females contain early stage egg chambers produced from germ-line and somatic stem cells [4]. While subsequent activity of these cell types is suppressed during diapause, the transcription level of genes with female reproductive functions can be detected relative to male expression, representing the stage of development when egg production arrests. Within the female-biased dataset, hierarchical clustering of genes by each time-point provides a measure of global expression similarity between samples (Figure 2b). It is clear that diapausing females in particular contain elements markedly differentiating this time point from the others.

Indeed, while there is evidence of a rise and then fall in the number of sexually dimorphic genes after the release of diapause (Figure 2a), vitellogenic samples retain the most similar global patterns of gene expression and cluster closely together (Figure 2b). Using diapause as a reference for pre-vitellogenic stages of development, we examined the transcriptional variation in females progressing into the primary period of egg production (q < 0.001, 2-fold). We find that through the cycle, 1922 transcripts accumulated to levels at least two-fold, and 864 transcripts to levels at least three-fold, higher during diapause [see Additional file 2]. Even though a subset of female-enhanced transcription during diapause will concern the early stages of egg development, other components of the expression profile will also be linked to maintaining a diapausing state and developmental arrest, which is discussed below in more detail.

#### a) Suppressed oogenic activity

**Figure 3:**
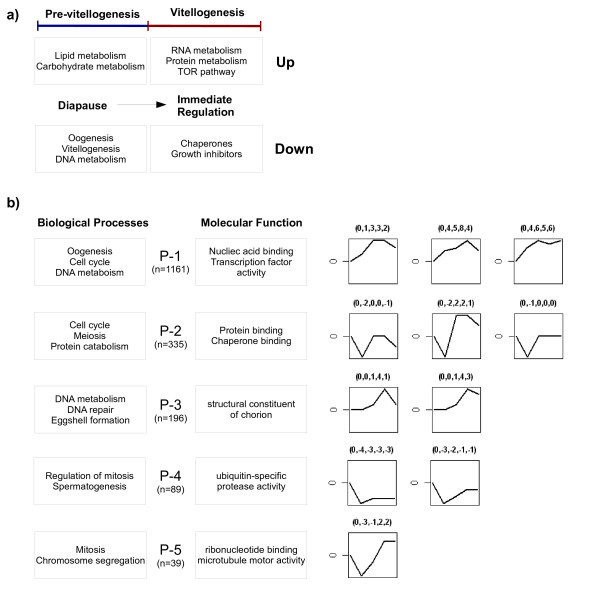
**Gene ontology summary and co-expression profiles**. **a) **Gene ontology summary of diapause and early vitellogenic regulation, featuring the highest over-represented Biological Processes annotations within each group. **b) **Gene ontology summary and co-expression clusters of oogenic biased expression. Boxes feature the highest over-represented Biological Processes and Molecular Function annotations within each expression profile (P1-P5). The total number of over-represented functional annotations for each cluster are provided [see Additional file 16, 17, 18, 19, 20]. Clusters were generated with Short Time-series Expression Miner (STEM, [56,57]). At each time-step genes were considered up or down regulated when compared to male controls. Expression profiles with a correlation of at least 0.8 were clustered together, generating five groups [see Additional file 21, 23]. Each cluster shown is over-represented at a P-value < 0.05 in permutation tests. Numbers in brackets represent the relative gene expression changes to which profiles were clustered at each point in the time series.

Of the female-biased transcripts detected during diapause, 356 are subsequently up-regulated after release from the restrictive temperature, indicating that these transcripts are largely repressed during this period (q < 0.001). Prior to reproductive arrest many aspects of oogenesis have been initiated in females. Several components of pre-vitellogenic egg chamber development are apparent, including expression associated with 'oogenesis', 'oocyte differentiation', 'follicle cell egg chamber development' and 'vitellogenesis'. A full list of repressed female-biased processes according to gene ontology annotations are provided (Figure 3a; [see Additional file 3, 4]).

In the early stages of oogenesis, many cellular processes are necessary for oocyte formation. Oogenesis in insects with meriostic ovaries is facilitated by the high degree of polyploidy in cells responsible for egg assembly and, prior to vitellogenesis, successive mitotic and modified cell cycles occur in the nurse and follicle cells [29]. A large number of transcripts repressed during diapause are linked with such functions, including activity of the 'mitotic cell cycle' as well as functions of 'DNA metabolism' and 'DNA replication' being among the most significantly represented groups.

Yet another important component of egg development repressed during diapause is the accumulation of yolk proteins (YP). Despite confirming that after 5 days in diapausing conditions females contain egg chambers from Stages 1–7, female-biased expression of primary yolk protein constituents were detectable (*Yp1*, *Yp2*, *Yp3*). Entry into vitellogenesis begins with increased production and incorporation of yolk protein precursors in the ovaries, however, these tissues are not the only site of yolk protein synthesis, since the female fat body also produces large quantities of YPs. As products synthesized by the fat body start to accumulate in the hemolymph immediately after eclosion, this tissue is the most likely source of vitellogenin expression in our dataset [30]. Following the release of diapause, all three YP transcripts are up-regulated and expressed at high levels throughout the time-series.

#### b) Immediately regulated transcripts

Genes with immediate changes in expression after entry into vitellogenesis may be directly involved in the regulation of developmental arrest and to explore this we identified those genes with a peak of activity at 24 hours that did not subsequently change significantly through the time-series (q < 0.001, 2-fold). The resulting set of early regulated genes consisted of 368 up-regulated and 381 down-regulated transcripts (Figure 3a; [see Additional file 5, 6]).

Consistent with an increase in cellular activity during the oogenic cycle, genes encoding 'protein metabolic processes' and 'RNA metabolic processes' are immediately induced. These annotations were enriched for regulators of 'ribosome biogenesis', including multiple members of the TOR signalling pathway (n = 16) [31]. TOR-dependent transcription is partly responsible for controlling growth rates and building the machinery for protein synthesis in eukaryotes. The efficiency of translation initiation is also an important correlate of cell growth and we find genes encoding for 'transfer-RNA metabolism' regulated immediately after diapause. In particular, the growth inhibitor *Thor*, which acts to prevent translation initiation, is down-regulated during this period [32].

In several insect species, a small group of transcripts from the heat-shock family of proteins are uniquely regulated during the oogenic cycle and are thought to have a role in regulating entry into vitellogenesis [6,33]. Heat-shock proteins are normally expressed in response to stress, acting as molecular chaperones to prevent the abnormal folding of proteins. During diapause, and in the absence of a general stress response however, the two heat-shock transcripts, *Hsp23 *and *Hsp70*, are up-regulated in females until exposed to normal environmental conditions. Once egg development has resumed, these transcripts then become down-regulated and production of a third transcript, *Hsp83*, is increased.

We also find an over-representation of 'protein folding' activity in the genes immediately regulated after diapause. One possible reason for the expression of heat-shock proteins is that as molecular chaperones the integrity of metabolic enzymes in over-wintering periods is improved. Certainly, expression of some of these genes is associated with the cold hardening response of flies [34]. However, another possibility is that they are involved in the regulation of diapause. Ecdysone, for example, directly regulates transcription by binding to the *ecdysone receptor*/*ultraspiracle *complex, and at least *Hsp83 *is also associated with the activation of this pathway [35].

As mentioned previously, the endocrine system plays a critical role in regulating many reproductive processes and entry into diapause is usually the result of a drop in hormone titres. Blocking Juvenile Hormone (JH) production, as occurs in most cases of adult diapause, results not only in the arrest of egg maturation but also induces physiological adaptations to prepare for over-wintering [6]. Increased rates of JH hydrolysis are then expected during diapause, and we find, consistent with this expectation, that JH catabolic activity is transcriptionally up-regulated during reproductive arrest.

As we note above, unlike most forms of adult reproductive arrest, yolk protein gene transcription is detected prior to the release of diapause in *D. melanogaster*. While JH is, in part, responsible for inducing many reproductive changes during development, the deposition of egg chamber yolk protein is coincident with increasing ecydsteroid levels [12,13]. In our oogenesis dataset, transcript levels of the *ecdysone receptor *(*EcR*) are not regulated significantly through the oogenic cycle, however *ultraspiracle *(*usp*) shows an increase in expression after the release of diapause. To date, *EcR *is the earliest response gene detected in pupal diapause, and increased activity has been recorded within an hour of returning flesh flies (*Sarcophagacrassipalpis*) to normal developmental activity [36]. In contrast, the other member of the receptor complex, *usp*, has a much longer response time under the same conditions, displaying a unique mode of activity in comparison to its molecular partner. Regulation of expression was also detected for an additional member of the early ecdysone response hierarchy after diapause, *broad*. As part of the early ecdysone genetic response, this gene has been implicated in the progression of many developmental processes, including the onset of vitellogenesis [37,38].

Suppression of growth, an important characteristic of diapausing insects, is controlled by the endocrine system, and in particular the insulin-signalling pathway. Inhibition of insulin production is critical for regulating the metabolic switch to lipid storage in some diapausing insects. Females destined for diapause in the mosquito *Culex pipens *normally regulate transcription of the components within this pathway, including the insulin receptor (*InR*) and the *forkhead *(*foxo*) transcription factor. Suppression of *InR *promotes activation of *forkhead*, prompting lipid build-up in females and ultimately ovarian arrest [39]. The insulin-signalling pathway has previously been implicated in the progression of oogenesis [40,41], as well as the ecological incidence of *Drosophila *reproductive diapause [9,42]. Similarly, we find regulation of *forkhead *transcription, among the 'lipid metabolic processes' regulated immediately in response to diapause.

#### c) Response to diapause

Insects that enter into photoperiodic diapause are expected to display patterns of expression specific to the mechanisms maintaining developmental arrest. During diapause, 564 transcripts are uniquely biased toward females and we believe they represent such functions (Figure 3a; [see Additional file 7, 8]). Insects entering into a state of reproductive arrest suppress growth and regulate metabolic pathways ensuring that there are enough energy reserves for prolonged periods of dormancy. Thus we expect that transcripts representing metabolic processes should display a shift towards conserving energy reserves. Indeed, in addition to the range of lipid metabolic processes regulated immediately after entry to vitellogenesis, we also find over-representation of 'lipid metabolism' functions within the set of female-biased transcripts, including a novel modulator of the insulin/PI3K pathway, *melted *[32].

By reducing TOR activity (translation) and activating *forkhead *(gene expression), *melted *interacts within the insulin-signalling pathway to mimic the effects of nutrient deprivation [32]. Consistent with these expectations, genes implicated in the starvation response of *Drosophila *were regulated within this dataset (n = 13) [43] as well as immediately after the release from diapause (n = 30) [43]. While many of the genes influenced by *melted *mutations are metabolism related, a significant number are also associated with proteolysis [32]. Transcription of genes encoding a variety of proteolytic enzymes was found to be highly regulated during diapause, the majority of which encode serine proteases, trypsin and carboxypeptidase activity with enriched expression in the midgut (n = 36/56).

At temperatures below 11°C, little digestive activity is expected in *Drosophila*, and while the production of digestive enzymes is directly regulated by food availability, in some insect species the build up of protease occurs prior to feeding. A suite of digestive enzymes, for example, are synthesized and stored in mosquitoes during reproductive arrest and these are subsequently utilized in the initial steps of digestion [44]. Unique digestive transcripts were regulated immediately upon activation of egg development in the early response genes described above. In particular, 6 members of the Jonah gene-family, highly enriched in midgut tissue, increased in activity and may play an important role in the regulation of digestive activity following diapause [45,46].

Lipids are the major form of energy storage in insects. Accumulation of triacylglycerols within specialized fat body tissues for example act to ensure that there are enough reserves for overwintering conditions, a process which occurs in several temperate *Drosophila *species preparing for further photoperiodic diapause [47]. However, even though slowing the use of energy reserves is crucial to the survival of diapausing insects, carbohydrate metabolism is an important function for fuelling basal metabolic processes, which typically becomes more apparent once an insect progresses deeper into diapause [48]. Here, we detect regulation of such activity within diapausing females and our dataset includes an over-representation of both 'lipid metabolic' and 'carbohydrate metabolic' functions. Some metabolic activity can be associated with a sophisticated level of adaptation to prepare for over-wintering conditions, and a common strategy in diapausing insects is to enhance resistance to environmental stress [6].

Tolerance to extremes of cold and desiccation can be improved by inducing a variety of physiological changes. For example, it is apparent that during diapause many 'structural constituents of the cuticle' are over-represented in our dataset. By decreasing permeability of the cuticle, evaporation rates and water loss can be reduced, which is critical for continued survival in harsh environmental conditions [49]. Such physiological changes are expected to employ molecular pathways that utilize, rather than store, energy reserves [50]. A number of 'carbohydrate metabolic processes' that are likely to contribute to physiological changes, including genes with 'chitin binding activity', were found to be biased in expression.

Many of the transcripts uniquely expressed in diapausing females are likely to have a role in longer-term adaptation to over-wintering conditions and expression levels may be expected to change slowly once egg development continues. Gene regulation during diapause itself is a dynamic process that we were unable to capture in detail with the single data-point used to characterized expression before entry into vitellogenesis. It is expected that while some genes will be up-regulated throughout diapause, others will be expressed for only a short period of time, including genes limited to expression during the early stages of induction, later once diapause is due to terminate, or even in a cyclically dependent fashion. Future research to understand the molecular basis of developmental arrest in *Drosophila *would benefit from an experimental design specifically taking these aspects into consideration.

#### d) Constitutive & abundant expression in oogenesis

The most abundantly expressed transcripts apparent during the time-series represent another interesting set of genes. These transcripts were identified by determining the cumulative abundance of transcripts in the top 15th percentile with the strongest signals and most constitutive expression patterns [see Additional file 9, 10]. Most of the resulting 197 transcripts correspond to functions associated with 'cellular biogenesis' and 'metabolic processes'. Indeed, transcripts related to protein synthesis, including translation initiation factors and ribosomal proteins, are particularly abundant. Functions related to 'cytoskeletal organisation' are also among the most strongly represented annotations. A variety of Tubulin and Actin encoding transcripts are members of this group, consistent with the need for ubiquitous rates of expression and participation in the mitotic cell cycle expected for such proteins. As expected, we find the primary constituents of 'vitellogenesis' are among the most highly expressed transcripts in the experiment, including the three primary yolk protein molecules (*Yp1*, *Yp2*, *Yp3*) and vitellogenin receptor, *yolkless*. We also find very high levels (up to 50-fold induction) of genes required for eggshell production, including components secreted by the somatic follicle cells, the vitelline membrane (*Vm26Aa*, *Vm26Ab*, *Vm34Ca*) and chorion membrane (*Cp15*, *Cp16*, *Cp18*, *Cp19*, *Cp36*, *Cp38*). High levels of eggshell gene transcription is facilitated by genome amplification in the follicle cells during the early stages of egg development [29]. Members of the chorion egg shell and vitelline membrane families are known to be expressed solely in the ovary, and serve as further validation of the datasets ability to detected genes expressed within this tissue.

### Clusters of gene expression

It is well known that female reproductive genes are typically well conserved components of the genome that are highly expressed in the ovaries [51]. Certainly, both the sex and tissue in which a gene is expressed influences the degree of sequence level variation apparent in a species [52-55]. However, not all genes expressed during egg development are female-biased in expression and similarly many may be preferentially expressed in other parts of the body. In order to identify biological and evolutionary processes underlying gene expression after diapause and during vitellogenesis, we have analysed the primary co-expression profiles apparent in our dataset using various sources of biological information [see Additional file 11, 12, 13, 14, 15, 16, 17, 18, 19, 20, 21, 22, 23].

Given the small number of time points in our experiment, we have clustered expression patterns on the basis of an algorithm specifically designed for short time-series analysis, whereby changes through the cycle are assigned to discrete blocks of expression [56,57]. At each time-step, genes are considered up or down regulated as compared to male controls. However, it must be noted that with this approach not all transcripts are assigned to significantly represented clusters and here we only consider those profiles with an over-represented number of genes. Expression profiles with a minimal correlation of 0.8 were clustered together, and in total, transcript patterns fit broadly into five groups (Figure 3b).

The three largest clusters, P1-3, represent almost 80% of gene expression within the dataset and several characteristics typical of egg production are apparent in these profiles. Once diapause is terminated, a major shift in the patterns of gene expression is expected with rapidly increasing growth rates and initiation of further development. Not surprisingly, over-represented GO annotation within P1 and P2 reveals a strong association with genes involved in 'oogenesis', 'cell cycle' and 'DNA metabolism' (Figure 3b; [see Additional file 16, 17]). Many basic cellular processes are necessary for the proper formation of oocytes and in non-diapausing females there is an abundance of genes related to progression of the cell cycle and supporting cellular metabolic machinery. P3 also contains genes involved in many aspects of 'cell cycle' and 'DNA repair' among other cellular functions; in addition, 'eggshell formation' transcripts were represented in this group (Figure 3b; [see Additional file 18]).

**Figure 4:**
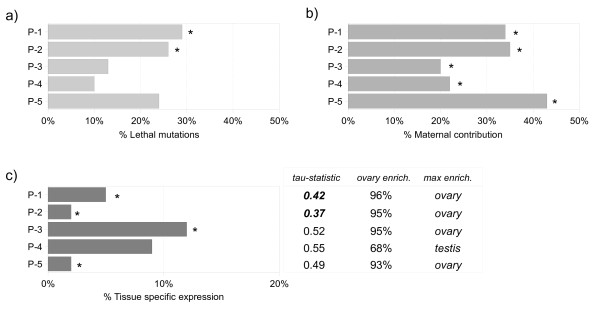
**Database summary of the primary oogenesis regulated profiles**. **a) **Lethal mutations. Proportion of genes annotated as 'Phenotypic class: Lethal'. **b) **Maternal contributions. Proportion of genes with maternal expression according to Hooper et al., 2007 [3]. **c) **Tissue-Specific. The number of genes with expression in a single tissue according to the FlyAtlas database. tau-statistic: 1= Specific; 0 = Ubiquitous (See Methods for further detail). Ovary Enrichment: The percentage of genes with higher ovary expression compared to whole-organism expression. Most Enriched Tissue: Tissue with the highest number of enriched expression versus whole-organism estimates. Expected values were determined from genome-wide estimates. Asterisks or numbers highlighted in bold were significant at the P < 0.05 level.

Concordant with this, the genetic dissection of *Drosophila *oogenesis has shown that many genes disrupting oogenesis often have indirect requirements or pleiotrophic effects linked to functions performed prior to adulthood. Mutation in such genes can result in reduced viability [58] and genes involved in egg development should reflect these characteristics. In addition to a low number of genes with tissue-specific transcription and a higher incidence of ubiquitous expression than expected, the genes within profiles P1 and P2 also have a high proportion of annotated lethal phenotypes (Figure 4a).

Another important characteristic of genes expressed during oogenesis is that they are likely to be a part of the regulatory cascades influencing early stages of embryonic development, since early embryogenesis is largely directed by maternally transcribed mRNA. As maternally deposited transcripts only become translationally active after fertilization, genes encoding such transcripts can be classified on the basis of distinctive expression profiles recorded during embryogenesis. An analysis of transcription from 30 time-points through the first 24 hours of development has identified a set of maternally derived genes in *Drosophila *[3], which we compared to the oogenic time-series.

The majority of regulated profiles were enriched for maternal products, supporting the notion that these genes are strongly linked to egg development (Figure 4b), and as a whole, we find 603 genes that are annotated as maternally contributed to the embryo. All maternal genes start with high levels of relative transcription, but subsequently show either a sharp decline "early" (3–5 hrs) or "late" (11–20 hrs) during the time-course. Of those transcripts in the present dataset, we find an equal representation of genes displaying early (n = 145/331) and late (n = 278/534) changes in expression. Among other vital functions, maternal constituents are responsible for initiating the replication and expression of the zygotic genome. Analysis of associated GO annotations reveals there is an over-representation of genes involved in 'cell division', 'DNA metabolism' and 'chromosome organization' functions that most likely facilitate the rapid cell divisions that take place in the early embryo [see Additional file 22].

### Evolutionary considerations

**Figure 5:**
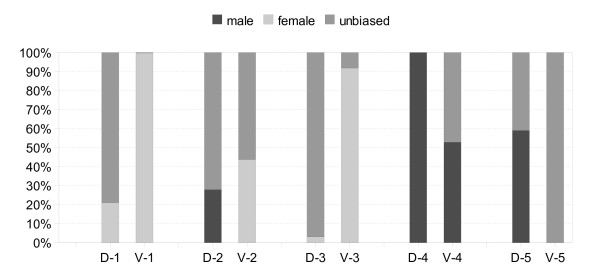
**Gender-biased expression ratios within co-expression clusters**. Expression profiles (1–5) before (D) and after (V) entry into vitellogenesis are shown. Gender-biased expression was calculated as genes with a 2-fold greater level of expression in either sex (q < 0.05), while unbiased expression was calculated as the remainder.

It has been reported previously that the gender in which a gene is expressed often acts to influence sequence level variation within and between species [55]. A large proportion of transcripts expressed either in the germline, or dependent on the presence of the germline, are gender-biased [51,59]. We find that in addition to enrichment for female-biased expression in P1 and P3, the majority of genes are also preferentially expressed in the ovaries (Figure 4c). However, it is apparent that not all profiles are expressed most abundantly in females when compared to males, including a smaller subset of profiles that remain either largely unbiased or at particularly elevated levels in males through the time-series (Figure 5).

We explored evidence for different rates of evolution using whole genome sequence divergence. Thanks to recent sequencing efforts, estimates of positive selection (ω) in models comparing non-synonymous (dN) to synonymous (dS) divergence rates are widely available for single orthologues in the *Drosophila *species complex that covers ~15 million years of evolution [60]. In addition to estimating the degree of evolutionary constraint across the phylogeny with a simple model of codon substitution (M0), we also calculate positive selection in gene profiles on the basis of site-specific models (M7, M8). These data are summarized in Table 1.

**Table 1:** Rates of sequence divergence across six *Drosophila *species.

	P-1	P-2	P-3	P-4	P-5
dN M0 (E:0.15)	0.13	0.12	**0.19**	**0.21**	0.15
ω M0 (E: 0.09)	**0.07**	**0.06**	0.10	0.10	0.09
					
ω M8 (E:10%)	85 (10%)	23 (9%)	14 (10%)	7 (11%)	5 (19%)

Expected values (E) were determined from genome-wide estimates. Figures represented in bold were significant at the P < 0.05 level.

While female reproductive transcripts occasionally evolve under positive selection [61], most reports indicate that these genes are influenced primarily by balancing selection [18-20]. Here, with exception of the two largest profiles in which divergence was lower than expected, positive selection of oogenic genes in the *Drosophila *phylogeny (ω) did not differ widely from whole genome expectations (Table 1). Overall, although some positive selection was detected in genes from the time-series, these instances were not over-represented and constituted only a small proportion of the genes detected.

In contrast, sequence variation of reproductive genes preferentially expressed in males is expected to show a high degree of evolutionary divergence [55]. Even though two of the five clusters in our dataset show little evidence of female-biased expression over the time-series, and an enrichment of gene expression in the testis (P4 +80:-10; P5 +25:-13), they do not appear to be evolving at faster rates (Table 1). As males and females have distinct roles in reproduction, evolutionary conflicts commonly arise when each sex is selected towards a different optimal phenotype. Certainly, intersexual fitness shows a substantial degree of discourse within *D. melanogaster *[62]. Genes with biased expression in the male accessory gland have received considerable attention in this regard, because when passed on to females during mating they can subsequently induce a variety of changes in female gene expression [63].

However, if a gene has either multiple functions or is expressed in multiple tissues, strong pleiotropic constraints may exist forcing selection to be sub-optimal in order to balance fitness requirements. Subsequent exploration of GO annotation indicates that many of these genes in P4-P5 have a role in 'mitosis', 'chromosome segregation' and 'spermatogenesis' (Figure 3b; [see Additional file 19, 20]), but while they are enriched for expression in the testis, these profiles also display a high level of ovary enrichment and are generally expressed throughout the body according to tau-statistics (Figure 4c; See Methods). Although elevated levels of these transcripts may then be required for sperm development, many are likely have more ubiquitous cellular functions, as well as being necessary for egg development.

## Conclusion

Our dataset provides further insight into patterns of gene expression during *Drosophila *oogenesis. Entry into vitellogenesis is an important stage of egg formation and by delaying entry past this control point, we have been able to investigate transcriptional dynamics before and after early egg development in the fly abdomen. Prior to reproductive arrest, many aspects of oogenesis have in fact been initiated in females. We detect transcription associated with several pre-vitellogenic developmental processes, including suppression of the cellular machinery necessary for successive mitotic and modified rounds of the cell cycle within early egg chambers. Yolk protein transcription is also largely inhibited during this period, however, once diapause is terminated by returning females to normal environmental conditions, a major shift in the patterns of gene expression is observed.

Immediately after introduction to normal environmental conditions, components most likely to regulate vitellogenesis are apparent, and we discuss evidence for the potential role of heat-shock proteins and hormone-regulated transcripts in this process. Even though *Drosophila *does not have the classic diapause response of many well adapted temperate insects, females display a shift towards regulating lipid reserves and inhibiting growth. Regulators of the insulin-signalling pathway that have previously been suggested to have at least partial control of developmental arrest are detected. Certainly, the correct nutritional cues are necessary for normal progression of the oogenic cycle and so may also have an important role in maintaining developmental arrest. Our data provide a baseline for the further exploration of diapause with the identification of gene expression changes immediately following release from oogenic arrest. The identified molecular processes, both specific to the ovary and at the whole animal level, provide an entry point for examining the physiology of this complex biological response.

Almost 14% of genes in the genome are developmental activated during vitellogenesis, the majority of which can be classified into discrete blocks of co-expression. All profiles contain genes with known or predicted roles in egg development, including an abundance of maternal constituents with important roles later in embryogenesis. Clusters with the largest number of maternal transcripts also display an over-representation of known lethal mutations and appear to evolve at normal or even reduced rates compared with estimates for the whole genome. It is generally expected that genes displaying sex-biased transcriptional abundance are sex-biased in function, however, not all profiles were preferentially expressed in females. We identify a smaller subset of profiles that remain either largely unbiased or preferentially expressed in males. Despite evidence for preferential expression in the testis and a role in spermatogenesis, these profiles were not evolving faster than the genome-wide estimates of divergence as might be expected.

Our studies provide a step towards further understanding the genetic control networks underlying egg development in *Drosophila*, and entry into vitellogenesis in particular. Analysis confirms many of the characteristics expected for genes involved in egg development, but also reveal a number of expression groups with unusual characteristics, including genes with apparent male-biased functions. Once genes with unique expression patterns during diapause are removed, 3358 transcripts were either female-biased in expression or significantly variable through the time-series and are proposed to have a role in egg development. Genes expressed during oogenesis are likely to make ideal candidates for the control of insect populations when transcripts that encode essential functions or are related to reproductive capacity can be targeted in population engineering programs. We hope the development of this regulatory framework will also enable comparative studies to be initiated with species whose control has important medical implications, such as the malaria vectors *Anopheles gambiae *and *Aedes aegypti*.

## Methods

### RNA collections and microarray platform

Microarray experiments employed the INDAC dual channel DNA microarray platform, containing 18,240 oligonucleotide probes (Gene Expression Omnibus (GEO) accession: GPL5016), prepared in the FlyChip microarray facility, University of Cambridge . Total RNA was prepared from samples of 10 abdomens, dissected under light CO_2 _aesthesia, by homogenization and extraction using TRIzol reagent (Invitrogen). Each RNA sample (5 μg) was initially reverse transcribed to cDNA before second strand synthesis was performed to obtain double stranded DNA. Double stranded DNA samples were then labelled with Cy3- and Cy5-fluorescent dye using the Klenow fragment of DNA polymerase prior to competitive hybridisation to microarrays in a Genomic Solutions hybridisation station for 16 hours at 51°C. After hybridization, slides were washed, spun dry and scanned with 635-nm and 532-nm lasers using a Genepix 4000B scanner (Axon Instruments). Full protocols are available from the FlyChip website. For this study oligonucleotide probes and genes were mapped to Flybase version FB2008.06 . Microarray data has been submitted to the Gene Expression Omnibus under the accession series: GSE13303.

### Normalisation of microarrays

After imaging, spot-finding and quantification were performed using Dapple software [64]. Dapple image analysis returns foreground and background intensity values for each probe, along with an indicator of spot quality, which are used to weight further normalization and analysis of differential expression. The resulting datasets were analysed using the Bioconductor software environment [65].

Prior to the analysis of differential expression, background correction and normalization procedures were performed using the LIMMA package [66] to remove poor quality features and standardize arrays for statistical comparisons. Background correction is responsible for the removal of localized and non-specific signals on microarrays that can arise from a variety of sources, such as labelled deposits left after washing stages or ambient noise in the scanning equipment. One way of dealing with such variation is to filter out low intensity spots, where foreground expression is lower than, or equal, to background intensity. However, this approach inevitably leads to missing values among probes, influencing variance and downstream analysis of differential expression. The preferred alternative is to stabilize variance as a function of intensity, such that lower intensity spots are offset to have reduced variation in fold changes.

Here, we employ the *normexp+offset *(50) method for background correction, an approach that has been show to produce the lowest rates of false discovery [67]. In addition to background correction, normalization of variation within, as well between arrays, needs to be considered in two-colour microarray experiments. Of particular concern is how to control for the effects of variation in printing quality and dye incorporation on spot intensity. Print-tip loess normalization provides a well tested method for local normalization of M-values within separate arrays, while variation across arrays can be standardized with quantile normalization of intensity values [68]. The print-tip loess method was implemented by weighting normalization in relation to spot quality. All analyses were performed on log-transformed ratios.

### Differential expression & gene clustering

Linear modelling was used to assess the differential expression of probes by one-way analysis of variance as implemented in the LIMMA software package [66]. Estimates of sample variance were pooled using empirical Bayes to generate a moderated *t*-statistic and increase confidence of differential expression. As part of the model, probe quality weights were included in the estimation of *t*-statistics, ensuring probes with high background intensities were removed from further analysis. Estimates of differential expression were adjusted for multiple testing using the false discovery rate method [69]. Adjusted F-statistics were used to identify genes in male or diapause reference comparisons with variable expression at the given fold change and significance level in the main text.

Discrimination of expression profiles through the oogenic cycle was achieved with clusters generated by the Short Time-series Expression Miner (STEM) [56,57]. This algorithm uses a simplification strategy to categorize expression as a function of time, in which changes through the time-series are assigned to discrete blocks of transcription. Simplification methods have the benefit of reducing noise in the original dataset by decreasing overall dimensionality of expression, making this type of analysis more robust to noise [70]. Moreover, unlike most unsupervised methods of clustering in which the number of groups must be provided before analysis (e.g. *k*-means), STEM generates a series of potential profiles with respect to the direction and magnitude of expression, after which enrichment of clusters is determined by comparing the distribution of observed groups to those expected in a random permutation.

### Statistical power

Post hoc power calculations were based on t-tests (alpha = 0.05) to measure the difference between male and female control samples [71]. The effective size for power (Type I Errors) was calculated using standard deviation of intensity measures within the 50th percentile of genes with least variance. We found that with a 2-fold-change of expression between treatments our analysis was expected to detect 96.8% of differentially expressed genes. Similarly, Pearson correlation was determined to be R^2 ^= 0.983 across all treatments using intensity values for genes with a calculated variance, further indicating that there is not a high degree of variation between the replicates of our experiments. Gene expression measures obtained with the INDAC probe set and quantitative-PCR are known to be highly correlated [72-74].

### Database resources

#### a) Gene Ontology annotation

Gene Ontology annotations  were examined for each list of differentially expressed genes and are available in the supplementary information [75]. To identify over-represented GO categories (July 2008 Annotation), a hypergeometric test was implemented in R against a subset of common high-level and low-level annotations representative of different functions using the GOstats library from the Bioconductor software environment [65]. Over-representation of annotations were adjusted for multiple testing using the false discovery rate method [69].

#### b) Tissue specificity

Gene lists were further integrated with the tissue-dissected microarray database, FlyAtlas [76]. Two measures of tissue specificity from 11 adult tissues (brain, midgut, hindgut, head, crop, Malphigian tubule, ovary, testis, accessory gland, thoracic and abdominal carcass and thoracico-abdominal ganglia) were subsequently obtained to include; a) expression enrichment for tissues versus whole animal comparisons, b) the degree of tissue bias as measured by normalizing against maximal expression [77]. Tissue bias values, as measured by the t-statistic, fall within the range of 0 to 1, in which higher values indicate more tissue-biased expression, i.e. a value of 1 indicates expression in a single tissue, and a value of 0 expression in all tissues.

#### c) Lethality

Genes annotated as 'Phenotypic class: lethal' were extracted from FlyBase [78] and integrated with gene list from the oogenic time-series. In total, 1715 genes of the 13100 genes (13%) tested were considered as lethal.

#### d) Rates of sequence evolution

PAML codon-substitution model estimates of positive selection were obtained from a previously published analysis of whole genome divergence in six species of the melanogaster group (*D. melanogaster, D. simulans, D. sechellia, D. yakuba, D. erecta, and D. ananassae*) [60]. In addition to estimating the degree of evolutionary constraint across the phylogeny with the simplest model of codon substitution (M0), we also calculated positive selection in gene profiles on the basis of site specific models (M7, M8) using a 10% false discovery rate.

## Authors' contributions

DAB conceived and designed the study, performed the diapausing experiments, and wrote the manuscript. SR participated in the design of this study and helped to draft the manuscript. Both authors read and approved the final manuscript.

## Supplementary Material

Additional file 1 **Female-biased gene table**. Expression levels and gene tables of transcripts female-biased in expression. Click here for file

Additional file 2 **Vitellogenesis gene table**. Expression levels and gene tables of transcripts regulated during vitellogenesis. Click here for file

Additional file 3 **Suppressed egg development gene table**. Expression levels and gene tables of transcripts suppressed during diapause, later involved in egg production. Click here for file

Additional file 4 **Suppressed egg development GO annotation enrichment**. The output of over-represented GO annotation of transcripts suppressed during diapause later involved in egg production. Click here for file

Additional file 5 **Early regulation gene table**. Expression levels and gene tables of transcripts regulated early after the release of diapause. Click here for file

Additional file 6 **Early regulation GO annotation enrichment**. The output of over-represented GO annotation for transcripts regulated early after the release of diapause. Click here for file

Additional file 7 **Diapause induced gene table**. Expression levels and gene tables of transcripts induced during diapause. Click here for file

Additional file 8 **Diapause induced GO annotation enrichment**. The output of over-represented GO annotation for transcripts induced during diapause. Click here for file

Additional file 9 **Constitutive transcription/most abundant gene table**. Expression levels and gene tables of transcripts having the most constitutive expression. Click here for file

Additional file 10 **Constitutive transcription/most abundant GO annotation enrichment**. The output of over-represented GO annotation for transcripts having the most constitutive expression. Click here for file

Additional file 11 **Profile 1 gene table**. Expression levels and gene tables of transcripts from Profile 1. Click here for file

Additional file 12 **Profile 1 GO annotation enrichment**. The output of over-represented GO annotation for Profile 1 transcripts. Click here for file

Additional file 13 **Profile 2 gene table**. Expression levels and gene tables of transcripts from Profile 2. Click here for file

Additional file 14 **Profile 2 GO annotation enrichment**. The output of over-represented GO annotation for Profile 2 transcripts. Click here for file

Additional file 15 **Profile 3 gene table**. Expression levels and gene tables of transcripts from Profile 3. Click here for file

Additional file 16 **Profile 3 GO annotation enrichment**. The output of over-represented GO annotation for Profile 3 transcripts. Click here for file

Additional file 17 **Profile 4 gene table**. Expression levels and gene tables of transcripts from Profile 4. Click here for file

Additional file 18 **Profile 4 GO annotation enrichment**. The output of over-represented GO annotation for Profile 4 transcripts. Click here for file

Additional file 19 **Profile 5 gene table**. Expression levels and gene tables of transcripts from Profile 5. Click here for file

Additional file 20 **Profile 5 GO annotation enrichment**. The output of over-represented GO annotation for Profile 5 transcripts. Click here for file

Additional file 21 **Combined profile 1–5 gene table**. A file containing the gene tables for all the combined cluster profiles 1–5. Click here for file

Additional file 22 **Maternal transcripts GO annotation enrichment**. The output of over-represented GO annotation for Maternal transcripts. Click here for file

Additional file 23 **Expression level clustergrams for gene clusters P1-P5**. Figures of expression level clustergrams for gene clusters P1-P5. Click here for file
