# Supplementary material for: Gene expression during Drosophila melanogaster egg development before and after reproductive diapause
Source: BMC Genomics. 2009 May 24;10:242. doi: 10.1186/1471-2164-10-242 (PMC2700134; doi:10.1186/1471-2164-10-242)
Supplement: Additional file 23 — Expression level clustergrams for gene clusters P1-P5. Figures of expression level clustergrams for gene clusters P1-P5. [file 1471-2164-10-242-S23.pdf]

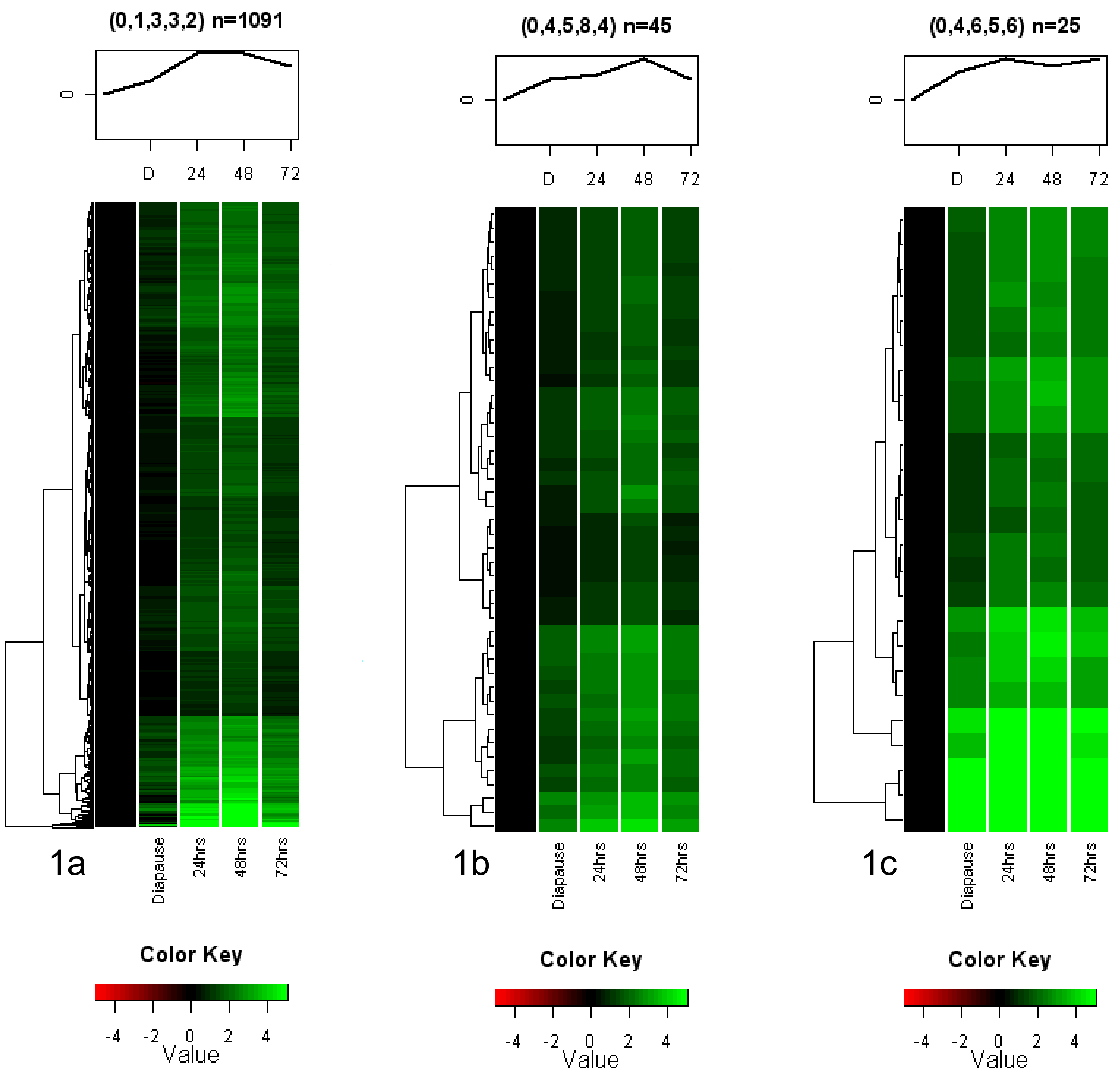
(0,1,3,3,2) n=1091
0
D
24
48
72
1a
Diapause
24hrs
48hrs
72hrs
Color Key
-4
-2
0
2
4
Value
(0,4,5,8,4) n=45
0
D
24
48
72
1b
Diapause
24hrs
48hrs
72hrs
Color Key
-4
-2
0
2
4
Value
(0,4,6,5,6) n=25
0
D
24
48
72
1c
Diapause
24hrs
48hrs
72hrs
Color Key
-4
-2
0
2
4
Value

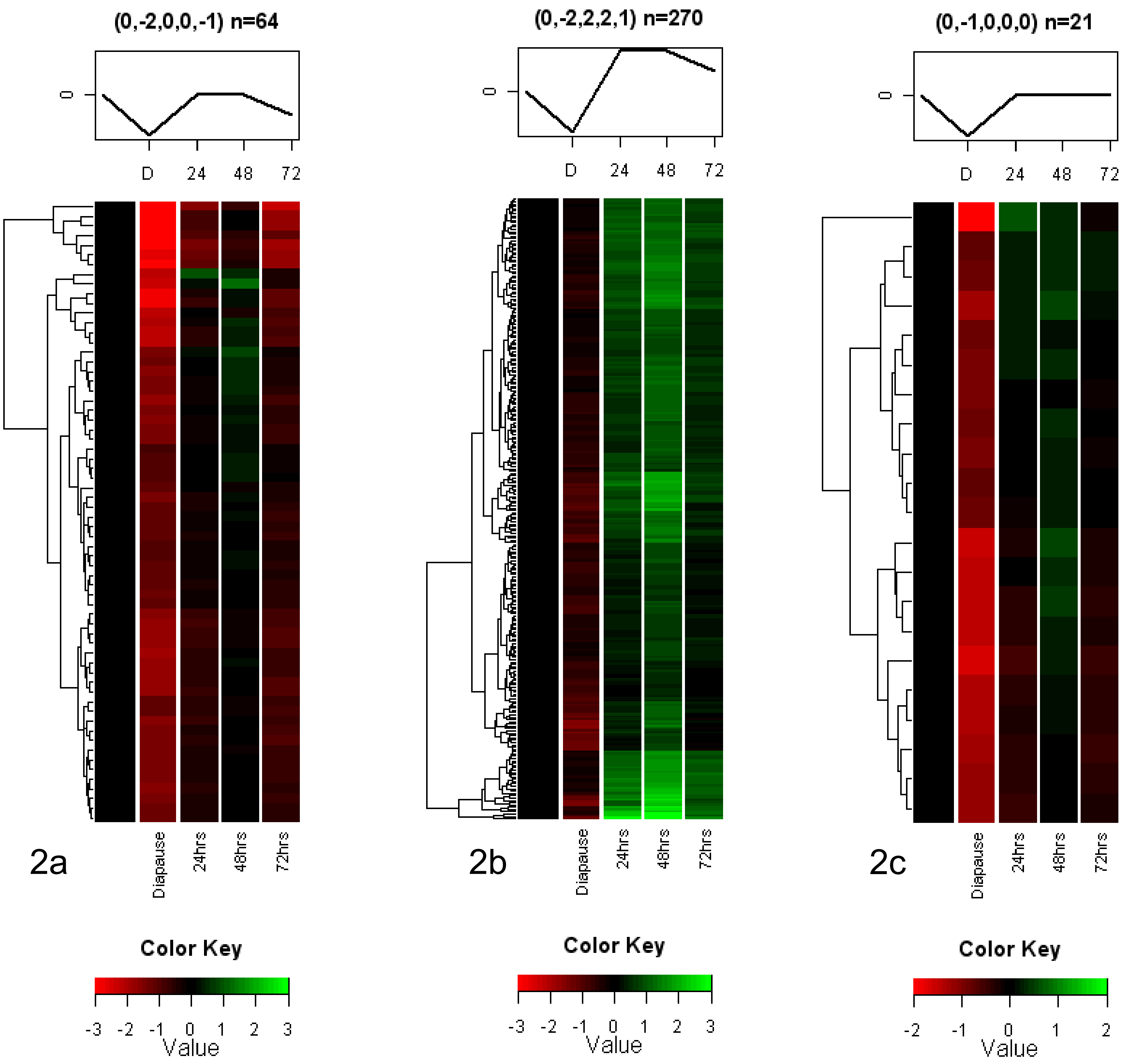
(0,-2,0,0,-1) n=64
(0,-2,2,2,1) n=270
(0,-1,0,0,0) n=21
0
D
24
48
72
Diapause
24hrs
48hrs
72hrs
2a
2b
2c
Color Key
Value
-3 -2 -1 0 1 2 3
-2 -1 0 1 2

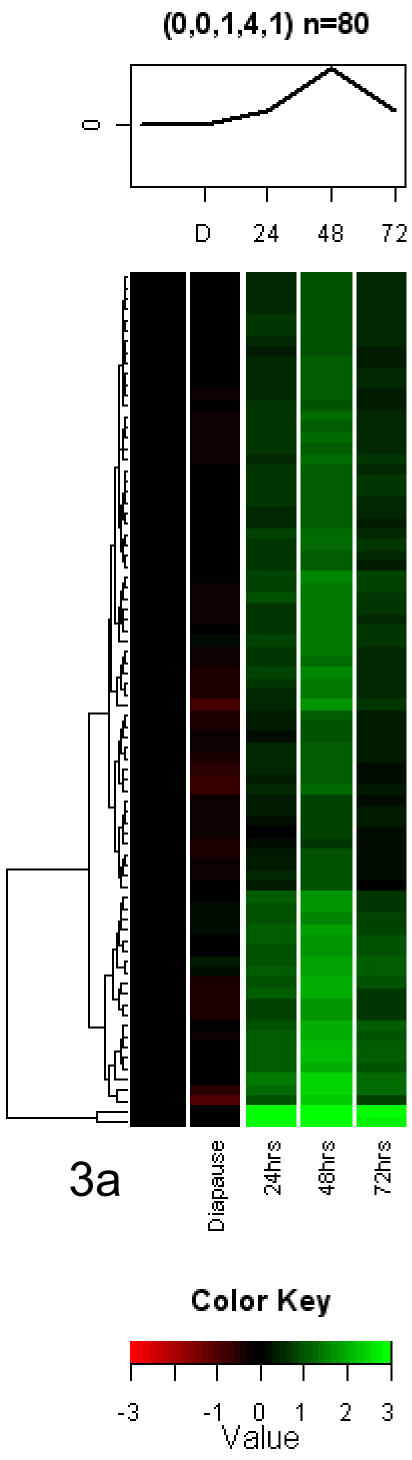
(0,0,1,4,1) n=80
0
D
24
48
72
Diapause
24hrs
48hrs
72hrs
3a
Color Key
-3
-1
0
1
2
3
Value

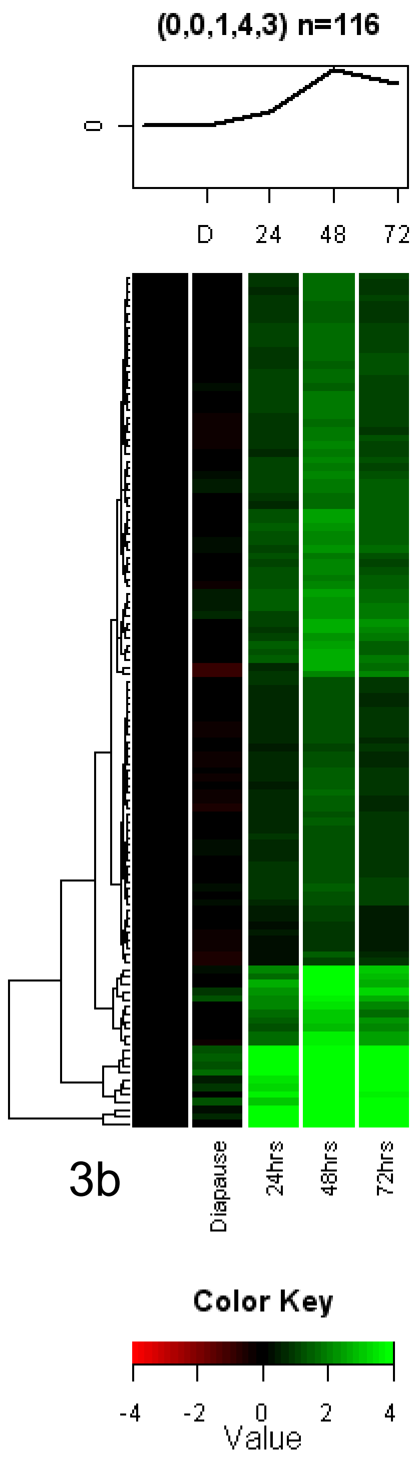
(0,0,1,4,3) n=116
0
D
24
48
72
Diapause
24hrs
48hrs
72hrs
3b
Color Key
-4
-2
0
2
4
Value

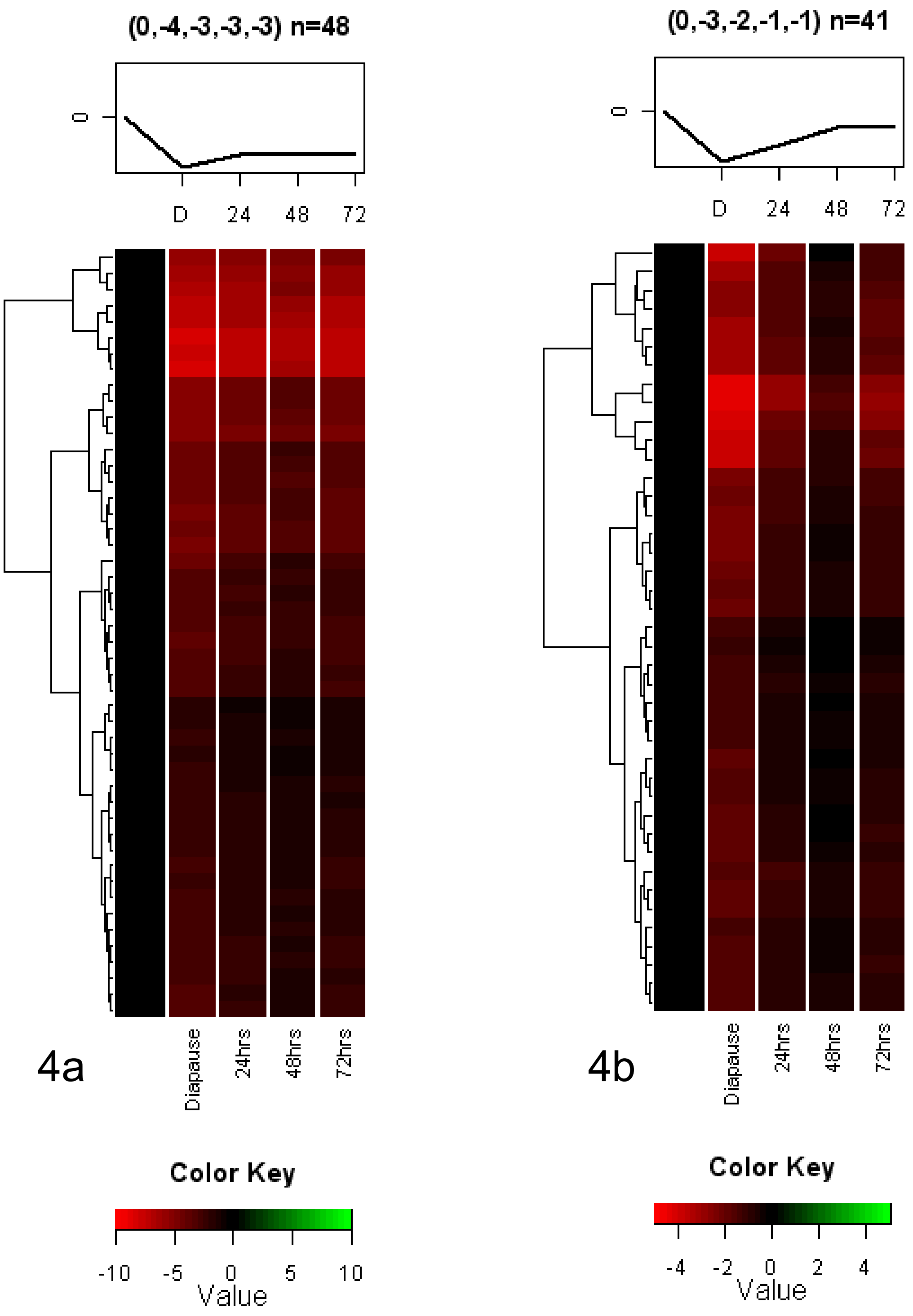
(0,-4,-3,-3,-3) n=48
0
D
24
48
72
Diapause
24hrs
48hrs
72hrs
Color Key
-10
-5
0
5
10
Value
(0,-3,-2,-1,-1) n=41
0
D
24
48
72
Diapause
24hrs
48hrs
72hrs
Color Key
-4
-2
0
2
4
Value

4a

4b

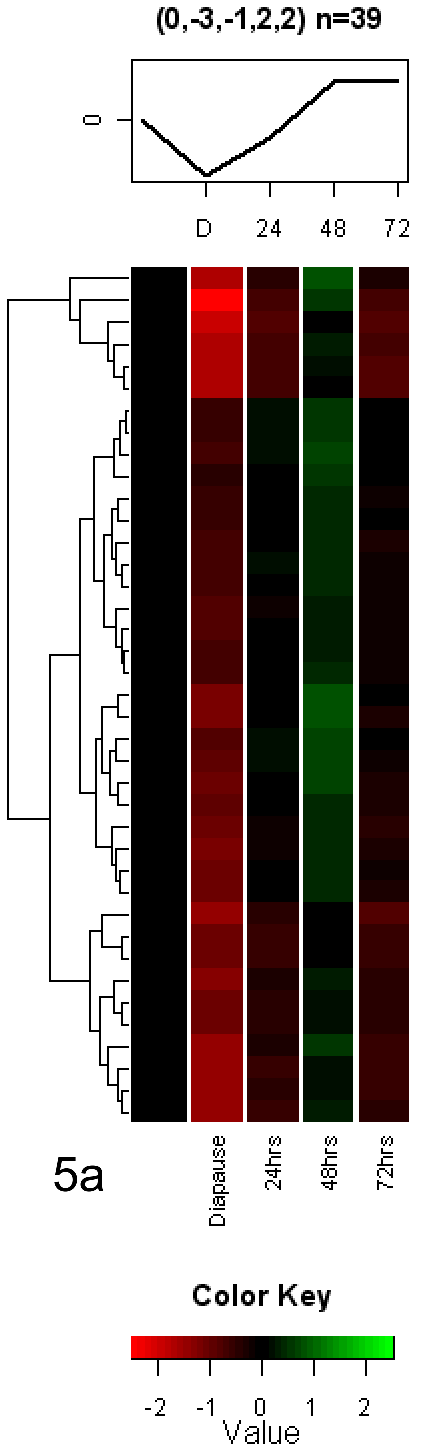

(0,-3,-1,2,2) n=39
0
D
24
48
72
Diapause
24hrs
48hrs
72hrs
Color Key
-2
-1
0
1
2
Value

5a
